# The Influence of Nutritional Factors on Verbal Deficits and Psychopathic Personality Traits: Evidence of the Moderating Role of the MAOA Genotype

**DOI:** 10.3390/ijerph121215017

**Published:** 2015-12-10

**Authors:** Dylan B. Jackson, Kevin M. Beaver

**Affiliations:** 1 Department of Criminal Justice, College of Public Policy, 501 W. Cesar E. Chavez Blvd, University of Texas at San Antonio, San Antonio, TX 78207, USA; 2 College of Criminology and Criminal Justice, 145 Convocation Way, Florida State University, Tallahassee, Florida, FL 32306-1273, USA; kbeaver@fsu.edu; 3 Center for Social and Humanities Research, King Abdulaziz University, Jeddah 21589, Saudi Arabia

**Keywords:** malnutrition, diet, food quality, meal deprivation, genetic risk, moderating effects, MAOA, males, verbal deficits, psychopathic personality traits

## Abstract

The current study explores whether: (a) nutritional factors among adolescent males predict their risk of exhibiting verbal deficits and psychopathic traits during adulthood and (b) the link between nutritional factors and these outcomes is conditioned by the MAOA genotype. The study analyzes data from the U.S. National Longitudinal Study of Adolescent Health (Add Health), a nationally representative, genetically informative sample. We find evidence that meal deprivation increases the likelihood of both verbal deficits and psychopathic personality traits, whereas poor quality nutrition increases the risk of verbal deficits. We detect the presence of a number of gene-environment interactions between measures of food quality and MAOA genotype, but no evidence of GxE in the case of meal deprivation. Limitations are noted and avenues for future research are discussed.

## 1. Introduction

Adequate nutrition is an essential component of healthy brain development [1]. Protein, iron, folate, essential fatty acids, and other key vitamins and minerals optimize the functioning of several brain areas, especially during the prenatal and early postnatal periods. Conversely, foods high in saturated fats have been shown to increase the risk of neuropsychological deficiencies by interfering with molecular substrates that aid numerous cognitive functions [2]. Ultimately, poor nutrition has the potential to impair learning/memory [3,4], reduce verbal ability [5,6,7], diminish executive functions [8,9], and interfere with modulators of synaptic plasticity, such as brain-derived neurotrophic factor (BDNF) [2,10]. Inadequate nutrition may also increase the risk of antisocial traits and behaviors. For instance, research has shown that poor nutrition during the early stages of the life course increases the likelihood of various criminogenic traits and behaviors during childhood and adolescence, including ADHD [11], externalizing behaviors [12,13], and aggressive/delinquent behaviors [14]. Psychopathic individuals, moreover, tend to exhibit brain deficits [15] that are often directly or indirectly influenced by the adequacy and quality of nutrition [4].

Although several studies have suggested that certain dimensions of nutrition influence both cognitive and behavioral outcomes, much of this work has been limited to childhood nutrition [3,7,9]. The tendency to emphasize childhood nutrition is understandable, given the substantial plasticity of the brain during this stage of the life course. Recent research, however, has shown that nutritional factors (e.g., fatty acids) during adolescence and adulthood can impact subsequent executive functioning [16,17], verbal ability [16,18,19], mood [20,21], and behavior [20,22]. It is also possible that the relationship between poor nutrition and neuropsychological and mental health outcomes may be moderated by genetic factors. Nevertheless, empirical tests of this possibility are generally lacking (except see [23,24]), despite a wealth of research indicating that gene-nutrient interactions influence the development of various physical ailments, including cancer and obesity [25].

In light of these voids in the literature, the goal of the present study is twofold. First, we test whether indicators of poor and inadequate nutrition during adolescence significantly increase the likelihood of two correlated outcomes during adulthood: verbal deficits and psychopathic personality traits [26,27]. Our second objective is to examine whether the link between nutritional factors, verbal deficits, and psychopathic personality traits is moderated by a functional polymorphism in the promoter region of the monoamine oxidase A (MAOA) gene, a polymorphism that has been linked to a host of antisocial outcomes [28,29].

### 1.1. Poor Nutrition, Verbal Deficits, and Psychopathic Personality Traits

A large body of research indicates that nutrition plays an important role in key brain functions [3,4,6,9,12,16]. Many of these studies have detected significant associations between nutritional factors and various components of verbal intelligence [6,19,30], including verbal fluency [3,5,7], word recall [31], and receptive vocabulary [32,33]. Furthermore, it appears that both diet quality [6,19,30] and severe malnutrition [5,7] can influence verbal ability across multiple stages of the life course. For example, using a sample of 1269 Chinese children, Liu and colleagues [30] found that children who regularly consumed breakfast scored higher on tests of verbal intelligence during the kindergarten school year. Similarly, a study by Gale and colleagues [6] revealed that children who regularly eat healthy foods, including fruit, vegetables, and home-cooked meals, exhibited higher verbal IQ scores at age 4 than children who infrequently ate healthy foods.

Some studies have also suggested that the benefits of a nutritious diet on verbal skills may not be limited to childhood. For instance, a recent study by Waber and colleagues [7] examined a sample of middle-aged, Barbadian adults and found that those who experienced moderate to severe malnutrition during infancy scored significantly lower on verbal fluency and associated cognitive tests, implying that the neuropsychological risk incurred through the experience of early nutritional deficits may persist for decades. Other research has revealed that a poor quality diet during the adolescent years is predictive of reduced verbal intelligence through early adulthood, even when genetic factors are taken into account [19]. Finally, research suggests that higher consumption of fatty fish during late adulthood enhances various aspects of verbal memory, including word recall [31]. Thus, the literature to date tends to detect a significant relationship between nutritional factors and various indicators of verbal ability.

Although very few studies have examined the link between poor nutrition and psychopathic personality traits (except see [34]), a number of studies have indicated that poor nutrition is significantly associated with a variety of mental health problems, including depression [35], psychosomatic complaints [14], schizophrenia [35], and schizotypal personality disorder [36]. Despite the paucity of research linking nutrition to specific dimensions of psychopathy, some research has shown that certain personality traits associated with psychopathy, including disinhibition, hostility, and aloofness, can be linked to diet quality [37].

A number of studies have also linked nutritional factors to an important correlate of psychopathic personality traits: violence [38]. Several researchers have randomly assigned study participants to receive micronutrient supplements to see whether doing so reduces violence and other problematic behaviors [20,39]. Results have generally been supportive of the link between ingesting supplements intended to mimic a well-balanced diet, reductions in violent and delinquent behaviors, and overall improvements in behavioral control. To illustrate, a study by Schoenthaler and Bier [39] found that, relative to controls, children whose diet was supplemented with key micronutrients (e.g., calcium, magnesium, and vitamin C) exhibited a 47% reduction in the rate of antisocial behaviors. Non-experimental studies have also indicated that consuming inadequate and poor quality food can heighten the risk of various behavioral problems during childhood and adolescence [12,13]. In light of this body of research, it is reasonable to suggest that nutritional factors might influence the development of psychopathic personality traits by early adulthood, particularly since verbal deficits and psychopathic personality traits are significantly correlated.

### 1.2. MAOA Genotype, Verbal Deficits, and Psychopathic Personality Traits

Genetic factors also appear to play an important role in the development of both verbal deficits and psychopathic personality traits [40,41,42]. For instance, a handful of studies have detected significant associations between the X-linked gene known as MAOA and diminished verbal ability [43,44,45]. In particular, males who possess a low-activity allele (*i.e.*, a 2 or 3-repeat allele) on MAOA are more likely to evince greater deficits in verbal and social communication skills [43,44,45], particularly among males who have been diagnosed with autism spectrum disorders. Additional research has found that MAOA interacts with other polymorphisms (e.g., COMT) to predict verbal intelligence among males with attention-deficit hyperactivity disorder [41]. Males who carry a low activity allele are also at greater risk of exhibiting aggression and violence relative to other genotypes [28,29].

Not surprisingly, MAOA genotype has also been linked to personality traits associated with psychopathy [42,46]. For example, Williams and colleagues [42] found that males with a low activity allele scored significantly higher on an index of various psychopathic personality traits (e.g., callousness, grandiose sense of self, pathological lying, disinhibition) relative to their high-activity MAOA counterparts. A study by Fowler and colleagues [46] garnered similar results using a sample of adolescents with a history of ADHD. The results of their study indicated that allelic variation on MAOA was significantly associated with total psychopathy scores as well as “emotional dysfunction” psychopathy scores (*i.e.*, callousness or lack of emotional responsiveness) in this high-risk sample. Research to date, therefore, suggests that allelic variation on MAOA is significantly predictive of neurological, behavioral, and personality differences in males, with carriers of a low-activity allele being particularly prone to negative outcomes [42,46,47].

### 1.3. Are The Effects of Poor Nutrition on Verbal Deficits and Psychopathic Traits Conditional?

While studies have detected significant associations between inadequate nutrition, verbal deficits and poor mental health outcomes, a few studies have also suggested that the strength of such associations may be contingent on other factors, such as sex [48] and parenting style [49]. Even so, very few moderators of the link between nutrition, verbal deficits, and mental health outcomes have been examined. For example, research has almost entirely ignored the possibility that genetic factors could moderate the relationship between nutrition, verbal deficits, and mental health. The few studies that have done so have typically examined neurological disorders associated with elderly populations (e.g., Alzheimer’s disease), rather than young adult populations [23,24]. Still, these studies suggest that the influence of nutrition on neurological health is likely contingent on allelic variation on important genetic markers.

Gene-nutrient interactions would be expected to influence neuropsychological and psychological outcomes, since essential nutrients as well as several candidate genes (e.g., MAOA, DRD2) often have overlapping influences on the synthesis of neurotransmitters in key brain structures. For example, both tryptophan, a dietary precursor to serotonin, as well as 5HHTLPR influence aggression/impulsivity by altering serotonergic functioning [50,51]. Georgieff [1], moreover, explains how a diet deficient in iron can alter monoamine neurotransmitter synthesis, which, at a genetic level, is also regulated by allelic variation on several genes [52]. The degree to which an individual’s neuropsychological or behavioral profile is substantially altered by patterns of nutritional input may therefore depend on their genotype, particularly on polymorphisms that have been linked to neuropsychological and behavioral abnormalities, such as MAOA.

### 1.4. The Current Study

In this study, we explore the possibility that the influence of poor nutrition on verbal deficits and psychopathic personality traits among males is conditioned by allelic variation on MAOA. Research has suggested that the effects of MAOA on negative outcomes are moderated by exposure to various noxious environments, including maltreatment [29] and maternal stress in utero [53]. Furthermore, a recent animal experiment [52] suggested that low activity MAOA genotype interacts with exposure to low levels of dietary iron in the womb to predict poorer cognitive functioning and maladaptive social behaviors (including aggressive temperament) in offspring. Still, whether the MAOA interacts with nutritional factors to predict verbal abilities and psychopathic personality traits among a human sample remains unknown. The present study extends this line of research and utilizes a large sample of youth to examine the direct and interactive effects of adolescent nutritional deficiencies and MAOA genotype on verbal deficits and psychopathic personality traits during adulthood.

## 2. Method

### 2.1. Sample

Subjects come from the U.S. National Longitudinal Study of Adolescent Health (Add Health). The Add Health is a prospective, nationally representative study that covers 14–15 years of development across adolescence and adulthood, making it well-suited to our research question. Furthermore, the Add Health also includes a number of participants (approx. 2500) who were asked to provide samples of their DNA for genotyping at the third wave of data collection. To be precise, buccal swabs were obtained for each of these participants so that their genetic profile on various candidate genes (e.g., DRD2, DRD4) could be determined [54].

In the case of MAOA, which is an X-linked gene, females have two copies of the gene, whereas males only have one copy. Therefore, interactions between MAOA and other risk factors must be examined separately for males and females, due to the distinct genetic profile of males and females on this gene. Much of the research on MAOA to date has linked low activity alleles to various antisocial and criminogenic outcomes in males [29,55]. As a result, we follow the lead of prior research using the Add Health [55] and restrict our sample to genotyped males (*N* = 1357).

### 2.2. Measures

#### 2.2.1. Outcome Measures

*Verbal Deficits*. Verbal deficits were measured using the Peabody Picture Vocabulary Test (PPVT). The PPVT involves an examiner showing various pages of pictures to the participant, with each page containing four distinct pictures. The examiner then speaks a word that describes one of the pictures presented and asks the subject to identify the picture that corresponds to the spoken word. The PPVT has been found to be a reliable and valid measure of verbal intelligence [56] that highly correlates with other indicators of cognitive ability [57]. For the purposes of our study, we utilized the PPVT percentile scores obtained at wave 3 (early adulthood). Furthermore, in order to create a measure of deficits in verbal ability, the original PPVT percentile scores were recoded so that a higher score reflects relatively poor verbal ability. Table 1 includes the descriptive statistics of the verbal deficits measure as well as all other variables and scales included in the analysis.

**Table 1:** Descriptive Statistics for the ADD Health Genotyped Subsample of Males.

Variable	Mean	Standard Deviation	Range
Verbal Deficits	51.26	28.58	0–100
Psychopathic Traits (Wave 4)	0.00	0.44	−1.40–1.52
Meal Deprivation	0.21	0.22	0–1
Low Vegetable Consumption	1.02	0.78	0–2
High Fast Food Consumption	2.43	1.83	0–7
MAOA (Low Activity)	0.43	0.50	0–1
Age Wave 1	16.11	1.68	12.12–20.86
Race (Non-White = 1)	0.34	0.48	0–1
Low SES	0.22	0.41	0–1
Low Self-Control (Wave 1)	0.00	0.65	−1.50–2.93
Family Meals	4.70	2.44	0–7
TV Viewing (Hrs per Week)	17.13	15.76	0–80
Video Games (Hrs per Week)	4.52	8.15	0–60
Neighborhood Disadvantage	0.00	0.61	−0.73–2.35
Low Maternal Attachment	0.00	0.87	−0.41–7.38
Maternal Disengagement	0.00	0.74	−0.95–3.97
Low Maternal Involvement	0.03	0.14	0–1
Low Maternal Supervision	0.46	0.66	0–3

*Psychopathic Personality Traits*. At wave 4, respondents were asked a number of questions that tapped their overall temperament and personality. Following the lead of prior research [58], we employed several of these items, taken from the five factor model of personality, to tap important dimensions of psychopathy, including lack of sociability, narcissism, low self-control, and callous/unemotional traits. We constructed a scale of psychopathic personality traits by standardizing and summing 23 relevant items from this personality inventory (α = 0.83). Items included questions about the degree to which participants lose their temper, have frequent mood swings, have little empathy/sympathy for others, rely on their impulses, and enjoy taking risks. Scores on each of the items ranged from 1 (strongly agree) to 5 (strongly disagree). Ultimately, all of the items included in the index were coded so that higher scores on the index reflected a more pronounced tendency toward psychopathic personality traits.

#### 2.2.2. Nutrition and Genetic Measures

*Meal Deprivation*. Elements of nutrition that can impact neuropsychological and mental health outcomes include both the sufficiency of food [59] and the quality/combination of foods [2]. For our first measure, we created an item that taps the extent to which subjects were failing to eat regular meals during adolescence. At the second wave of data collection, subjects were asked three questions regarding how frequently they ate breakfast, lunch, and dinner during the past week, with response options ranging from 0 (zero days during the past week) to 7 (seven days during the past week) for each of the three items. Scores on each item were reverse coded so that higher scores reflected less frequent consumption of each meal during the previous week. The reverse-coded scores on each of the three items were subsequently summed and divided by the total number of possible meals during the previous week (*i.e.*, 21). Consequently, the meal deprivation item represents the proportion of total meals during the previous week that were skipped, with scores closer to 1 indicating greater meal deprivation. We should note that, while the concept of meal deprivation is similar to the concept of food insufficiency, they are not interchangeable. For instance, studies examining malnourished children define food insufficiency as “an inadequate amount of food intake due to a lack of money or resources” [59]. The current measure of meal deprivation, however, cannot definitively determine the reason underlying the infrequent eating of regular meals (*i.e.*, whether it was elective or not). Nonetheless, our measure is an improvement in terms of specificity of change in food intake, as it captures gradual decreases in regular meal consumption. Prior research on children has generally classified food insufficient households as those that “sometimes or often do not get enough food to eat” [59], which cannot capture the possibility of a dose-response relationship.

*Low Vegetable Consumption*. In addition to our measure of meal deprivation, we included two measures of food quality: low vegetable consumption and high fast food consumption. During wave 1 of data collection, respondents were asked a small number of questions regarding their eating habits. We followed the lead of prior research [60] and utilized a question about the number of times the youth ate vegetables during the day prior to the interview. Response options ranged from 0 (did not eat) to 2 (ate twice or more). In order for our item to reflect low vegetable consumption, this item was reverse-coded so that subjects who reported eating vegetables less frequently received higher scores.

*High Fast Food Consumption*. A high frequency of fast food consumption has been linked to poorer mental and physical health outcomes [61], and thus may be predictive of the outcomes in our study. For our second measure of food quality, we followed the lead of prior research [62] and employed an item from the second wave of data collection that asked about the frequency with which adolescents ate fast food during the previous week. Examples of fast food were given during the interview, such as McDonald’s, KFC, Pizza Hut, and Taco Bell, in order to avoid misclassification. Response options for this item ranged from 0 (zero days during the previous week) to 7 (every day during the previous week).

*Monoamine oxidase A (MAOA)*. As mentioned previously, genetic information on a number of polymorphisms was obtained for approximately 2500 subjects at the third wave of data collection. An X-linked gene known as MAOA was one of the polymorphisms examined in the genotyped subsample of the Add Health study. Previous research has linked this gene to various indicators of maladjustment in males, including impulsivity [47], violence [29], and gang involvement [28]. Specifically, males who possess low activity alleles (i.e., the 2 or 3 repeat allele) incur a greater risk of developing these negative traits and behaviors. In light of this body of research, and in line with prior research using the Add Health [28], MAOA was coded so that males possessing the 2 or 3 repeat allele were assigned a value of 1, whereas males possessing higher repeat alleles (*i.e.*, the 3.5-repeat allele, the 4-repeat allele, or the 5-repeat allele) were assigned a value of 0. As displayed in Table 1, roughly 43% of the sample possessed a low activity allele, whereas the remainder of the sample possessed a high activity allele.

### 2.3. Adolescent Traits

*Low Self-Control*. At the first wave of data collection, participants were asked a number of questions tapping their tendency toward impulsivity. An impulsive temperament is more likely in individuals with poor nutrition [63], and has also been found to be closely tied to both neuropsychological deficits [64] and psychopathic traits [65]. At wave 1, adolescents were asked a number of questions regarding their level of self-control, including whether they go with their gut feeling when making a decision, think about the consequences of their decision, gather ample facts in order to solve a problem, try to think of alternative solutions to a problem, and analyze what went right or wrong after taking a particular course of action. The five items were coded so that higher scores reflect lower levels of self-control. An index was then created by standardizing and summing the recoded items (α = 0.66). Importantly, our measure of low self-control is informed by Hirschi’s [66] recent conceptualization of low self-control, which he describes as “the tendency to consider the full range of potential costs [and long-term implications] of a particular act”. Furthermore, the items included in our scale also reflect the recent findings that suggest a close nexus between executive dysfunction, poor decision making, and low self-control [67].

### 2.4. Family Socialization, Home Environment and Neighborhood Measures

*Family Meals*. A number of studies have suggested that the frequency of family meals has a significant influence on adolescent nutrition [68]. It is possible, moreover, that the structure provided by family meals may influence adolescent criminogenic outcomes as well [69]. As a result, we include a measure that taps the frequency of family meals in our study. At wave 1 of data collection, adolescents were asked how many days during the past week they ate at least one of their meals with their parent/parents. Response categories ranged from 0 (no days during the past week) to 7 (every day during the past week).

*TV Viewing*. We also included a measure of TV viewing, as high levels of TV viewing have been associated with poorer diet [70] as well as antisocial traits and behaviors [71]. At the first wave of data collection, youth were asked to estimate how many hours a week they spent watching television. Although nine participants reported more than 80 h of television a week, these subjects were coded as 80 ha week to maintain a more reasonable range of values (80 h a week would require an average of more than 11 hours a day of TV viewing).

*Video Games*. In addition to television viewing, a measurement of the frequency of video game participation was included. Recent research has shown that playing video games, along with other sedentary behaviors, is associated with a poorer diet [72]. Some research has also suggested that frequent video game participation is associated with antisocial tendencies [73]. Consequently, we include a measure of video game participation in our study which taps the number of hours spent playing video games in the past week. Scores on this item range from 0 to 60, with higher numbers reflecting greater exposure to video games.

*Neighborhood Disadvantage*. We also create a measure of neighborhood disadvantage using six items [74]. At wave 1, youths were asked whether they were happy in their neighborhood, whether they knew most of their neighbors, whether they felt safe in their neighborhood and whether they wanted to move. Items were recoded to reflect greater displeasure with (and concern about) the neighborhood. Subsequently, items were standardized and summed to construct the index (α = 0.66).

*Low Maternal Attachment*. We also followed the lead of prior research [75] and constructed an index of maternal attachment at wave 1. Respondents were asked how close they felt to their mother and how much they thought their mother cared about them, with responses ranging from 1 (not very close/much) to 5 (very close/much). Items were coded so that higher scores were indicative of lower maternal attachment. The index was created by standardizing and summing the items (α = 0.67).

*Maternal Disengagement*. In line with prior research [74], we created an index designed to tap the extent to which the subject’s mother was disconnected from her child’s life. At wave 1, youth were asked whether they were satisfied with their relationship with their mother and with the way she communicates with them, whether their mother is warm or loving toward them, and whether she calmly corrects their mistakes and misbehaviors. Responses to the items were standardized and added together to create an index in which higher scores indicate more maternal disengagement (α = 0.79).

*Low Maternal Involvement*. We also developed an item assessing low maternal involvement. At wave 1, adolescents were asked if they had engaged in a number of activities with their mother during the past four weeks, including playing a sport, attending church, shopping, going to a movie or special event, and talking about school or a personal problem. Adolescents who reported participating in none of the activities with their mother were assigned a value of 1, whereas adolescents who reported participating in at least one of these activities with their mother were assigned a value of 0.

*Low Maternal Supervision*. Finally, a measure of maternal supervision was created by using three items provided at wave 1 [76]. Participants were asked if their mother is typically home when they leave for school in the morning, arrive home from school, and go to bed. Subjects who responded “almost never” or “never” were assigned a value of 1 on each of the three variables; otherwise, they were coded as a 0. The recoded items were then added together so that subjects whose mothers provided the lowest amount of supervision scored the highest on this variable (final range of responses: 0–3).

### 2.5. Controls

*Age*. We also included a continuous variable in the analysis measuring the age of each respondent (in years) at wave 1.

*Race*. A measure of race (1 = nonwhite; 0 = white) was also included in the analysis in an attempt to rule out any confounding due to racial differences of the subjects.

*Low SES*. Low SES was measured using four items that asked caregivers about the whether they had received various forms of financial assistance at wave 1 [76]. To be precise, caregivers reported whether they had received food stamps, unemployment compensation, Aid to Families with Dependent Children, or public housing/housing subsidy within the month prior to data collection. Caregivers who reported having received any assistance within the previous month were assigned a value of 1, whereas caregivers who reported receiving no assistance were assigned a value of 0.

### 2.6. Plan of Analysis

Ordinary least squares (OLS) regression is used to test our hypotheses. Because our final sample included sibling pairs, standard errors may be deflated due to case non-independence. We followed the lead of prior research [77] and estimated Huber/White standard errors to account for the clustering of cases by family. Our analysis proceeds as follows. First, we examine whether indicators of poor food quality and meal deprivation during adolescence were significantly predictive of verbal deficits during early adulthood, net of controls.  Second, we test whether these same indicators of poor nutrition are predictive of psychopathic personality traits during adulthood. Most importantly, we estimate six regression equations exploring whether statistical interactions between MAOA genotype and our three indicators of poor nutrition (*i.e.*, meal deprivation, low vegetable consumption, high fast food consumption) are predictive of verbal deficits and psychopathic personality traits among males. Moderating effects were examined using multiplicative interaction terms between the nutritional factors and MAOA. Covariates were mean-centered prior to creating the interaction terms [78].

## 3. Results

Table 2 and Table 3 display the results of eight regression equations that estimate a) whether poorer nutrition during adolescence significantly increases the likelihood of verbal deficits and psychopathic personality traits during adulthood and b) whether MAOA moderates the association between indicators of poor nutrition and these outcomes.

**Table 2:** The Direct and Interactive Effects of Three Nutritional Measures and Low MAOA Activity on Verbal Deficits in Young Adult Males.

Covariates	Verbal Deficits			
	Model 1 b/β	Model 2 b/β	Model 3 b/β	Model 4 b/β
Meal Deprivation	1.85 *, 0.06 (0.87)	1.87 *, 0.06 (0.87)	1.84 *, 0.06 (0.87)	1.90 *, 0.07 (0.88)
Low Vegetable Consumption	4.64 *, 0.16 (0.87)	4.64 *, 0.16 (0.87)	4.64 *, 0.16 (0.86)	4.65 *, 0.16 (0.87)
High Fast Food Consumption	0.71, 0.02 (0.86)	0.72, 0.02 (0.87)	0.72, 0.02 (0.86)	0.79, 0.03 (0.86)
MAOA	−0.98, −0.03 (0.83)	−0.97, −0.03 (0.82)	−0.97, −0.03 (0.82)	−0.97, −0.03 (0.82)
Age	−0.97, −0.06 (0.55)	−0.97, −0.05 (0.56)	−0.93, −0.05 (0.56)	−0.99, −0.06 (0.55)
Race (non-white)	15.27 *, 0.25 (1.90)	15.34 *, 0.25 (1.90)	15.34 *, 0.25 (1.89)	15.41 *, 0.25 (1.89)
Low SES	14.53 *, 0.20 (2.18)	14.44 *, 0.20 (2.18)	14.60 *, 0.20 (2.16)	14.24 *, 0.20 (2.18)
Low Self-Control(Wave 1)	2.97 *, 0.07 (1.36)	2.92 *, 0.07 (1.35)	3.22 *, 0.07 (1.36)	2.84 *, 0.06 (1.34)
Family Meals	−0.50, −0.04 (0.36)	−0.52, −0.04 (0.36)	−0.49, −0.04 (0.36)	−0.51, −0.04 (0.36)
TV Viewing	0.11, 0.06 (0.06)	0.11, 0.06 (0.06)	0.10, 0.05 (0.06)	0.11, 0.06 (0.06)
Video Games	0.15, 0.04 (0.10)	0.15, 0.04 (0.10)	0.16, 0.04 (0.10)	0.16, 0.04 (0.10)
Neighborhood Disadvantage	−1.55, −0.03 (1.43)	−1.60, −0.03 (1.43)	−1.51, −0.03 (1.43)	−1.52, −0.03 (1.43)
Low Maternal Attachment	−2.29, −0.06 (1.33)	−2.27, −0.06 (1.33)	−2.18, −0.06 (1.30)	−2.23, −0.06 (1.31)
Maternal Disengagement	−1.56, −0.04 (1.34)	−1.53, −0.04 (1.34)	−1.53, −0.04 (1.33)	−1.38, −0.03 (1.35)
Low Maternal Involvement	4.27, 0.02 (8.96)	3.86, 0.02 (8.96)	4.00, 0.02 (8.79)	3.99, 0.02 (8.75)
Low Maternal Supervision	−0.06, 0.00 (1.31)	−0.14, 0.00 (1.31)	−0.16, 0.00 (1.31)	0.06, 0.00 (1.32)
Meal Deprivation X MAOA	NA	0.79, 0.03 (0.82)	NA	NA
Low Vegetable Consumption MAOA	NA	NA	2.21 *, 0.08 (0.84)	NA
High Fast Food Consumption X MAOA	NA	NA	NA	1.93 *, 0.07 0.85
*N*	1030	1030	1030	1030
*R*^2^	0.20	0.20	0.20	0.20

The number in parenthesis are standard errors. * indicates that *p* < 0.05.

**Figure 1:**
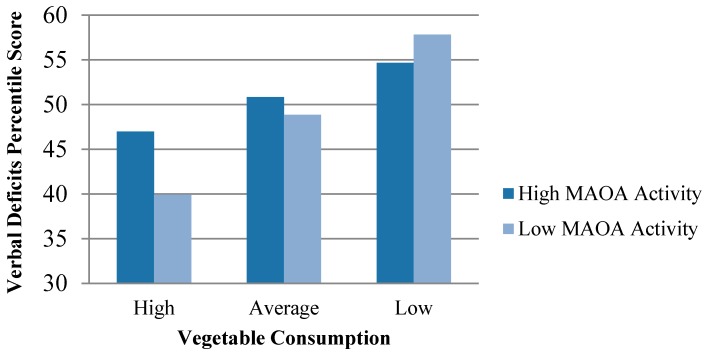
Verbal Deficits Percentile Scores by Level of MAOA Activity and Vegetable Consumption When Covariates are at their Mean.

**Figure 2:**
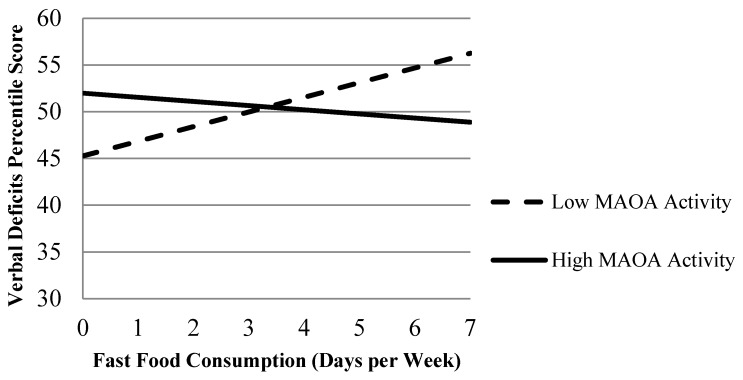
The Expected Effect of Fast Food Consumption on Verbal Deficits by MAOA Genotype When Covariates are at their Mean.

To be precise, the first model of each table examines direct effects, whereas models 2 through 4 of each table explore gene-environment interactions between elements of poor nutrition and MAOA. The results of the direct effect models indicate that subjects who frequently skipped meals during their adolescent years were significantly more likely to exhibit poorer verbal ability during early adulthood and psychopathic traits during adulthood, net of family, neighborhood, and demographic factors. We should note that, although meal deprivation significantly predicted both of these outcomes, the effect of meal deprivation on psychopathic personality traits was particularly sizeable (β = 0.17, the largest beta in the model). Nevertheless, despite the relevance of meal deprivation to the development of psychopathic personality traits, indicators of poor food quality had no significant direct influence on such traits. In the case of verbal deficits, however, food quality appeared to be more relevant. Specifically, low vegetable consumption during adolescence significantly increased the likelihood of verbal deficits during early adulthood (β = 0.16), although the effects of fast food consumption were null. The significant effect of low vegetable consumption is all the more noteworthy considering that none of the neighborhood or family socialization measures emerged as significant predictors of verbal deficits (see Model 1 of Table 2). We now turn to the results of our interactive models. Models 2 through 4 of Table 2 contain the results of 3 regression equations that examined whether each nutritional measure (*i.e.*, meal deprivation, low vegetable consumption, and high fast food consumption) interacted with the low-activity MAOA genotype to predict verbal deficits during early adulthood. Two of the three interactions emerged as positive and significant. In particular, the results suggest that subjects who consumed fewer vegetables and more fast food during their adolescent years were especially likely to exhibit verbal deficits during early adulthood if they also possessed a activity MAOA genotype (see Figure 1 and Figure 2). No significant interactions between meal deprivation and MAOA emerged, however, in the prediction of verbal deficits.

**Table 3:** The Direct and Interactive Effects of Three Nutritional Measures and Low MAOA Activity on Psychopathic Personality Traits Among Adult Males.

Covariates	Psychopathic Personality Traits			
	Model 1 b/β	Model 2 b/β	Model 3 b/β	Model 4 b/β
Meal Deprivation	0.07 *, 0.17 (0.02)	0.07 *, 0.17 (0.02)	0.07 *, 0.17 (0.02)	0.08 *, 0.18 (0.02)
Low Vegetable Consumption	0.01, 0.02 (0.02)	0.01, 0.02 (0.02)	0.01, 0.02 (0.02)	0.01, 0.02 (0.02)
High Fast Food Consumption	0.02, 0.04 (0.02)	0.02, 0.04 (0.02)	0.02, 0.04 (0.02)	0.02, 0.04 (0.01)
MAOA	0.02, 0.04 (0.01)	0.02, 0.04 (0.01)	0.02, 0.04 (0.01)	0.02, 0.05 (0.01)
Age	−0.01, −0.03 (0.01)	−0.01, −0.03 (0.01)	−0.01, −0.03 (0.01)	−0.01, −0.03 (0.01)
Race (non-white)	0.02, 0.03 (0.02)	0.02, 0.03 (0.02)	0.02, 0.03 (0.02)	0.03, 0.03 (0.03)
Low SES	0.11 *, 0.10 (0.04)	0.11 *, 0.10 (0.04)	0.11 *, 0.10 (0.04)	0.10 *, 0.09 (0.04)
Low Self-Control(Wave 1)	0.10 *, 0.15 (0.02)	0.10 *, 0.15 (0.02)	0.10 *, 0.15 (0.02)	0.10 *, 0.14 (0.02)
Family Meals	0.00, 0.00 (0.01)	0.00, 0.00 (0.01)	0.00, 0.00 (0.01)	0.00, 0.00 (0.01)
TV Viewing	2.4 × 10^−3^ *, 0.08 (1.1 × 10^−3^)	2.4 × 10^−3^ *, 0.08 (1.1 × 10^−3^)	2.4 × 10^−3^ *, 0.08 (1.1 × 10^−3^)	2.4 × 10^−3^ *, 0.08 (1.1 × 10^−3^)
Video Games	1.6 × 10^−3^, −0.03 (2.4 × 10^−3^)	−1.7 × 10^−3^, −0.03 (2.5 × 10^−3^)	−1.6 × 10^−3^, −0.03 (2.4 × 10^−3^)	−1.5 × 10^−3^, −0.03 (2.4 × 10^−3^)
Neighborhood Disadvantage	0.03, 0.05 (0.03)	0.03, 0.05 (0.03)	0.03, 0.05 (0.03)	0.04, 0.07 (0.02)
Low Maternal Attachment	0.03, 0.06 (0.02)	0.03, 0.06 (0.02)	0.03, 0.06 (0.02)	0.04, 0.07 (0.02)
Maternal Disengagement	0.04, 0.07 (0.02)	0.04, 0.07 (0.03)	0.04, 0.07 (0.03)	0.05 *, 0.08 (0.02)
Low Maternal Involvement	0.08, 0.02 (0.16)	0.07, 0.02 (0.16)	0.08, 0.02 (0.16)	0.08, 0.02 (0.16)
Low Maternal Supervision	−0.04 *, −0.06 (0.02)	−0.04 *, −0.07 (0.02)	−0.04 *, −0.06 (0.02)	−0.04 *, −2
Meal Deprivation X MAOA	NA	0.02, 0.04 (0.01)	NA	NA
Low Vegetable Consumption X MAOA	NA	NA	0.00, 0.01 (0.01)	NA
High Fast Food Consumption X MAOA	NA	NA	NA	0.07 *, 0.16 (0.01)
*N*	919	919	919	919
*R*^2^	0.12	0.12	0.12	0.15

**Figure 3:**
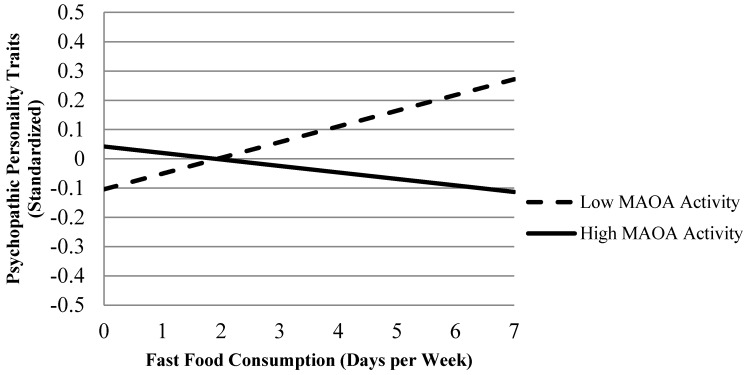
The Expected Effect of Fast Food Consumption on Psychopathic Personality Traits by MAOA Genotype When Covariates are at their Mean.

We also examined the role of the same gene-environment interactions in the development of psychopathic personality traits. Models 2 through 4 of Table 3 display the findings of three regression equations that explored whether the nutritional measures interacted with MAOA genotype to predict psychopathic personality traits during adulthood. Although not identical to the results in Table 2, the findings reiterate the relevance of food quality for the neuropsychological and mental health of males with a high-risk genotype on MAOA. Specifically, the significant, positive interaction shown in Model 4 of Table 3 indicates that participants who consumed a greater amount of fast food during their youth were at significantly higher risk of developing psychopathic personality traits by adulthood, but only if they possessed a high-risk allele on MAOA (see Figure 3; β = 0.15). Neither MAOA nor fast food consumption had significant, independent effects on psychopathic traits, but when examined interactively, they add to the predictive value of the model (*R*^2^ increased 25% with the inclusion of the interaction term). Allelic variation on MAOA, however, did not moderate the relationship between meal deprivation and psychopathic personality traits. Moreover, no significant interaction between low vegetable consumption and MAOA emerged when predicting psychopathic personality traits.

## 4. Discussion

Inadequate nutrition has been shown to predict a host of negative life outcomes, whether physical [25], psychological [35,36], or neuropsychological [3,4]. Empirical examinations of the role of dietary choices during adolescence in predicting neuropsychological and mental health outcomes during adulthood, however, are lacking in comparison to studies on childhood nutrition (except see [79]). Furthermore, research on neuropsychological and mental health outcomes often fails to explore how the influence of nutritional factors might vary as a function of allelic variation on key genetic markers (e.g., MAOA) (except see [23,24]). The primary aim of the present study was to address these gaps in the literature by testing whether features of adolescent nutrition are significant predictors of verbal deficits and psychopathic personality traits during early adulthood, and whether these relationships might be moderated by MAOA. Analysis of the Add Health data revealed three key findings.

First, the results revealed that both meal deprivation and low vegetable consumption during adolescence significantly increased the likelihood of verbal deficits during adulthood, whereas only meal deprivation significantly increased the likelihood of psychopathic personality traits. The findings are generally consistent with the research to date on the potentially deleterious effects of poor nutrition on neuropsychological functioning, even during later life stages [16]. However, the absence of any significant direct effect of poor food quality on psychopathic personality traits is somewhat unexpected considering diet quality has been linked to both violence [20] and negative mental health outcomes [35,36]. Still, our results, as a whole, highlight the role of both inadequate and poor quality nutrition in subsequent neuropsychological and psychological functioning.

Second, we found evidence that a poor quality diet during adolescence is especially detrimental to the adult verbal ability of individuals who possess a low activity allele on MAOA. To be precise, subjects who reported eating vegetables less frequently and fast food more frequently were particularly prone to verbal deficits in early adulthood if they carried the 2 or 3 repeat allele on MAOA. This finding speaks to the potential relevance of genetic factors in moderating the effects of poor quality nutrition on neuropsychological outcomes [52]. Our third and final finding is similar to the second. We found that psychopathic personality traits were significantly more likely among adult subjects who ate fast food more frequently during their youth, but only if they also possessed a low activity MAOA allele. The effect of high fast food consumption on subsequent psychopathic personality traits, therefore, only emerged once genotyped was considered and included as a moderator in the study.

## 5. Conclusions

While our study makes an important contribution to the literature, it is not without its limitations. First, it would have been useful to also test hypotheses using measures that tap specific nutrients (e.g., omega 3 fatty acids, iron), but such measures were not available in the data. Some of the recent literature utilizes very precise measures of nutrient intake and metabolization, such as omega 3 blood levels [34] and iron deficiency in utero [52]. Nevertheless, more generalized measures of poor diet are useful in highlighting the specific dietary changes that can be made on a daily basis to improve subsequent neuropsychological and mental health [6,9,19]. On a related note, it would have been preferable to have a greater number of diet items and more thorough measurements of diet (e.g., food journals/records). Second, a wider array of neuropsychological tests and diagnostic tools (e.g., fMRI) would have been preferable to test the robustness of our results. We were only able to measure impaired verbal ability and thus cannot directly assess the relevance of the results for other cognitive functions. Still, our verbal deficits models likely yield conservative tests of the influence of nutrition on neuropsychological functions, as other brain regions that are less relevant to verbal ability specifically may also be impacted by poor nutrition. Finally, the generalizability of our study is restricted due to the inclusion of a number of sibling pairs in our final sample. Nevertheless, recent research suggests that the findings of studies that examine samples of sibling pairs may be more generalizable to the broader non-sibling population than originally assumed [80].

In conclusion, poor dietary habits during youth appear to place individuals at risk of both verbal deficits and psychopathic personality traits during adulthood, particularly for those with a high-risk genotype on MAOA. Future research should seek to replicate and build upon the findings of our study by employing additional samples, more diversified and more thorough measures of nutrition, a broader array of candidate genes, and other measures of neuropsychological functioning. Additionally, research in this area should explore the relevance of nutrient-gene interactions in the prediction of outcomes associated with verbal deficits and/or psychopathic personality traits, including violence, offending, and criminal justice involvement. Doing so will elucidate ways in which families, school administrators, and other practitioners can intervene in order to improve the life outcomes of at-risk youth.
